# Supplementary material for: Feasibility of using an energy balance to measure evapotranspiration in green stormwater infrastructure
Source: PLoS One. 2021 Feb 3;16(2):e0246286. doi: 10.1371/journal.pone.0246286 (PMC7857564; doi:10.1371/journal.pone.0246286)
Supplement: Attachment — Submitted filename: Response to Reviewers.docx [file pone.0246286.s001.docx]

Dear Review Panel,

Thank you for your thorough review of our draft manuscript. We genuinely appreciate the feedback and we think that your review has helped us strengthen the manuscript. Below, we address specific questions and concerns of the reviewers. Throughout the manuscript we have made edits to improve language and justifications.

- **Has the statistical analysis been performed appropriately and rigorously?**

**Reviewer #1: Yes**

**Reviewer #2: No**

Response: We believe that the only appropriate statistical analysis for a 12-point data set has been carried out. We presented averages, standard deviations, and a graphic of the relationship between measured and calculated evapotranspiration. Any further statistical analysis would likely violate assumptions inherent in the analysis or at least accepted standards. We do understand that a more robust data set would be ideal. With the limited research funding we had, we believe this data set accomplishes the goal of determining whether or not energy balance methods should be further investigated for uses in GSI.

Reviewer #1

- **“…there are points in the manuscript that are not well justified such as lines 71-74 that are missing of references.”**

Response:. We went through and added references at this point, and throughout as we thought applicable. Some points are left without a reference as they are common thought within the industry.

- **“…I have an objection with the duration of the experiment and the number. Maybe another one for more accuracy is needed.”**

Response: The intent, going in to the summer of 2019, was to collect > 30 data points, however, due to equipment issues the study was delayed. The study could not continue late into the fall because of plant senescence and there was not funding available to continue this study in summer 2020. We agree that more investigation is needed to refine the method and more robustly make statements of the estimation relationship. At present, even though there is only 12 data points, the results from this feasibility experiment do demonstrate that the idea of estimating ET using thermal imaging deserves attention, which was the goal of the study.

- **“It is mentioned in the text that there are three equations that are being used for the ET. Are there? It is not clear enough.”**

Response: Thank you! The three reference ET equations are presented to compare to the mass balance ET calculated and to validate meteorological data. Then ET calculated via mass balance is compared to ET calculated via energy balance. The ways in which the equations are used for the analysis is discussed in lines 42-46 and 103-108. We added some clarification in lines 236-241 hoping to clear up any confusion on how each of the three *reference* ET equations were used. *Reference* ET is calculated via three different equations. *ET* is measured via the mass balance method and calculated via the energy balance method.

- **“The authors chose to use MATLAB. What is the reason for? Why not something else?”**

Response: There was familiarity within the department with MATLAB coding. Also, see added note on line 282 about MATLAB’s built-in image processing functions.

- **“Do the authors take into consideration that the camera is in a stable position and that may cause a shading and gives other results than the expected ones?”**

Response: Good question. This was considered and addressed early on in the experimental period. Figure 1 is misleading because it was taken before we realized that shadows cast on the Switchgrass plants but not over the solar radiation sensor would be problematic. Even though the experiments were carried out during the middle of the day, and interference from shadows was unlikely to happen, the camera and tripod were moved to a different position to avoid the potential issue after the second full data collection day (8/30/2019).

- **“In line 363 it is not clear how the soybean and the switchgrass are having the same view factors. Generally it is not well written and clear how the authors have chosen the view factors.”**

Response: Soybean and Switchgrass do not necessarily have the same view factors. We added some wording in lines 370-371, 378-379, and 379 to hopefully clarify that these values were only used as a starting point to investigate whether or not changes to the F_a_ and F­_g_ terms (if justified by further study) could make the energy balance more accurate. The purpose of the small analysis on the view factors is hopefully clear between the explanation in lines 369-371 and 374- 379.

- **“Moreover, are the conditions the same in order to have each lysimeter in a test position?”**

Response: We think this question is asking about the position of the camera over the lysimeters and whether or not that was consistent. We have added clarification on lines 152-154.

- **“In line 377, the statement needs a better justification.”**

Response: Line 377 was meant to reference the entire study. The entire study is our justification for the statement on line 377. In case line 377 could be interpreted to only be referencing the view factor investigation, we have moved the paragraph to the Summary section below (where it probably should have been in the first place) and added some clarification in line 396.

- **”In line 385, it is said that there is no need of calibration. I have an objection and it is expendient to be done a better search in the scientific bibliography.”**

Response: There was indeed no calibration done to any of the terms in the energy balance equation used to produce the main results of the study (Table 3). We didn’t say there is no need for calibration. Line 404 says “…useful results are obtainable without any calibration or scaling.” We are trying to say that even without calibration, reasonable results can be obtained.

- **“In line 428-429, the sentence causes a lot of questions about the experiment and the results.”**

Response: Good catch. We intended for the statement in lines 444-445 to refer to the reference ET equations only. This has been clarified in line 443.

- **“In conclusion, the manuscript needs an overall overview by the authors in order to explain in a better way many aspects in the text, especially the whole experiment, the lysimeters , the average height of the plant.”**

Response: We have added a note regarding the date the photo in Fig 1 was taken on line 107 and some clarifying details about the Switchgrass plants in lines 132-135. Overall we have revised the manuscript to ensure the experiment and results are well-explained.

Reviewer #2

i) **“Line 76: “arewidely” space is missing.”**

Response: Good catch! This has been addressed.

ii) **“Lines 81, 395-396: You could make “Green stormwater infrastructure” to be “GSI” as you do in other cases. I suggest to define the abbreviation GSI in the first mention (line 33) and then you can call it as GSI.”**

Response: The GSI abbreviation was defined in line 33 as suggested. And “Green stormwater infrastructure was abbreviated in lines 411-412, and other locations. However, we try to avoid starting sentences with abbreviations. We also redefined in line 58 in case a reader skips the abstract.

iii) **“Lines 84-85: “Energy balance methods have not been investigated to calculate ET for GSI.” You should probably re-examine or rephrase that, from a little search I did, I found a paper in which the Eddy-covariance method is investigated in green infrastructure. Here is the link of the paper:** [**https://www.sciencedirect.com/science/article/pii/S0048969716323567?via%3Dihub**](https://www.sciencedirect.com/science/article/pii/S0048969716323567?via%3Dihub)**”**

Response: Line 89 has been updated to reflect that thermal imaging has been used. There are a few studies on eddy covariance on green roofs, but we did not find on other types of GSI, such as bioretention, which is likely as EC systems generally are better where there is a monoculture and sufficient fetch. Additionally, EC provides longer-term averaging of ET (e.g., https://webcache.googleusercontent.com/search?q=cache:q1qnCNfaZ78J:https://stormwater.pca.state.mn.us/images/a/ab/Trees_Task_13_ET_interception_credits.docx+&cd=10&hl=en&ct=clnk&gl=us).

iv) **“Figure 1: I see that the heights of the plants in lysimeters are quite different. It is on purpose? If it was on purpose, what is the point? I think this requires more explanation. Having in mind the consideration of ASCE for tall reference crop, do you think this difference of the height between the three plants (if it’s real) influences the results? In lines 286-288 is referred “The average height of the Switchgrass plants in T1, T2, and T3 from August 29, 2019 to October 4, 2019 (0.60m)”, the average of three plants for the entire period (August 29, 2019 to October 4)?”**

Response: The plants were allowed to grow naturally without any restriction or trimming. They grew at different rates but ended up being approximately the same shape and size. The shape and size of all plants was nearly constant from August 29 to October 4. We have added these points to lines 132-135.

v) **Table 1: In the 3 columns of “Reference ET” units are missing.**

Response: This has been corrected.

vi) **“Lines 330-331: “Over the course of 12 data collection events, the average ET estimated by the EB was within 1 mm (or 32%) of the MB measured ET and there was no calibration done to any parameters in the EB.” I do not exactly understand what is the meaning here.”**

Response: We modified this sentence to clarify the meaning.

vii) **“Concerning the data set, do you think is large enough for the calibration of Fa and Fg coefficients requirements? Additionally, what about the validation of the energy balance model?”**

Response: We do not believe the data set is large enough for calibration of the F_a_ and F_g_ coefficients. We do not attempt to use our data for calibration of the view factors. Instead, we present a short investigation into the view factors to show that they have influence on the energy balance and formulating more accurate coefficients for typical GSI systems could lead to a more accurate energy balance. We think the question regarding validation would be taken up in a follow up study where we could calibrate components of the energy balance equation.

viii) **“Line 394: “An energy balance like the one described here may be the only practical way to measure ET in green stormwater infrastructure, and thus, the only way to fully resolve the water balance in these systems.” I am not sure about the meaning and the correctness of this.”**

Response: Lines 411-412 and 415 have been changed to help clarify because we understand that other energy balance methods may be available (like Eddy-covariance mentioned in a previous comment). We do hold, however, that energy balance methods (thermal imaging or otherwise) are the only practical way to fully resolve the water balance in GSI.

Please let us know if any further clarification is needed. Thank you for your help refining our paper!

Sincerely,

Devin Krasowski

Bridget Wadzuk
