# Supplementary material for: Feasibility of using an energy balance to measure evapotranspiration in green stormwater infrastructure
Source: PLoS One. 2021 Feb 3;16(2):e0246286. doi: 10.1371/journal.pone.0246286 (PMC7857564; doi:10.1371/journal.pone.0246286)
Supplement: Attachment — Submitted filename: Response to Reviewers.docx [file pone.0246286.s002.docx]

Dear Review Panel,

Thank you for your thorough review of our draft manuscript. We genuinely appreciate the feedback and we think that your review has helped us strengthen the manuscript, once again. Below, we address specific questions and concerns of the reviewers. Throughout the manuscript we have made edits to improve language and justifications.

Reviewer #2

- **“I read the original and the revision submission and I found that many corrections have been done and the responses to the reviewer were satisfactory. I have only one objection. Lines 441 – 442: “may be the only practical 441 way to measure ET GSI, and thus, the only way to fully resolve the water balance in these systems.” I think you could use some milder words (“only way”).**

Response: This comment is likely in reference to lines 412-414 in our second submission. Line 394 in submission 2 seems to be a blank line. We have removed the second “only” in that sentence to avoid confusion. However, we have left “..only practical…” in line 413 for the following reason. Consider Equation 1 in the manuscript. ET was able to be calculated as the residual of a water mass balance in our lysimeters because our experimental set up allowed us to track infiltration and weigh the lysimeters. Most GSI systems cannot practically be set up to track infiltration or to be weighed. Therefore, ET has to be estimated or measured via other means. The only methods we are aware of that are able to do this involve measuring or approximating the movement of energy. In addition, the first part of the sentence reads: “Energy balance methods**, like the one described here, may be…**” There we recognize other energy balance methods may suffice and entirely different practical methods **may** someday exist.

Reviewer #4

1) **“In Line 129, although the authors have pointed out that “Three lysimeters contained Switchgrass (labeled T1 through T3)”, I still do not understand what T1, T2, T3 mean. I think the authors should describe them clearly.**

Response: This has been addressed in line 129.

2) **“In Line 347-348, “the average ET estimated by the EB was within 1 mm (or 32%) of the MB measured ET without any calibration being done to parameters in the EB. This small difference.…..”. In fact, I think “32%” is not a small difference.”**

Response: We have updated lines 347 through 350. The difference from Table 3 is actually 4.12 – 3.16 = 0.96 mm, or 30%. This is comparable to the difference given from in Table 1 between the reference ET_0T_ and the MB ET (3.16 – 2.34 = 0.82 mm, or 26%). We do think this is a relatively small difference considering the simple approach taken with the energy balance for this feasibility study. Although it seems the reference ET equation performed better here with less effort, this was likely because the Switchgrass systems used in our study were uniform and similar to the tall reference crop assumed in the equation for ET_0T_. Most GSI are not uniform and/or violate other assumptions inherent to the reference ET equations. This is where we believe the advantage and transferability of physically measuring energy flux comes in.

3) **“In Line 394: “An energy balance like the one described here may be the only practical way to measure ET in green stormwater infrastructure, and thus, the only way to fully resolve the water balance in these systems.” I am not sure about the meaning and the correctness of this.**

Response: This comment is likely in reference to lines 412-414 in our second submission. Line 394 in submission 2 seems to be a blank line. Please see our response to Reviewer #2 Comment 1.

4) **“In Line 411-413: “Energy balance methods, like the one described here, may be the only practical way to measure ET in GSI, and thus, the only way to fully resolve the water balance in these systems.” As suggested by the former reviewer, I am also not sure about the meaning and the correctness of this. I think the method should not be the only way in GSI. Please consider and correct it.”**

Response: Please see our response to Reviewer #2 Comment 1.

Please let us know if any further clarification is needed. Thank you for your help refining our paper!

Sincerely,

Devin Krasowski

Bridget Wadzuk
