# Supplementary material for: Feasibility of using an energy balance to measure evapotranspiration in green stormwater infrastructure
Source: PLoS One. 2021 Feb 3;16(2):e0246286. doi: 10.1371/journal.pone.0246286 (PMC7857564; doi:10.1371/journal.pone.0246286)
Supplement: Attachment — Submitted filename: Response to Reviewers.docx [file pone.0246286.s003.docx]

Dear Review Panel,

Thank you for your thorough review of our draft manuscript. We genuinely appreciate the feedback and we think that your review has helped us strengthen the manuscript, once again. Below, we address specific questions and concerns of the reviewers. Throughout the manuscript we have made edits to improve language and justifications.

Reviewer #4

1) **“I still do not understand what T1, T2, T3 mean. Is there a difference among these three treatments? If yes, what is the difference? I think the authors still did not describe them clearly.”**

Please see the changes made on lines 129 and 131. The setups were identical, T1-T3 was just the chosen naming convention.

2) **”In line 347-351, whether the difference is small should be evaluated quantitatively by using statistical methods, rather than qualitative judgments.”**

Please see the changes made in lines 349-351. We believe we have evaluated this difference quantitatively and appropriately by comparing the percent difference given by our energy balance method with the percent difference given by the established reference ET equation (Equation 11).

Please let us know if any further clarification is needed. Thank you for your help refining our paper!

Sincerely,

Devin Krasowski

Bridget Wadzuk
